# Plot and landscape-level estimates of tree biomass and carbon stocks in Panama’s mangrove Important Bird Areas

**DOI:** 10.1038/s41597-025-05354-5

**Published:** 2025-06-17

**Authors:** Jorge Hoyos-Santillan, Alejandro Miranda, Juliana Chavarría, Carlos Hormazabal, Blas Mola-Yudego, Esperanza González-Mahecha

**Affiliations:** 1 Smithsonian Tropical Research Institute, Panama City, Panama; 2 Audubon Americas, New York, USA; 3 School of Biosciences, University of Nottingham, Sutton Bonington, UK; 4 School of Forest Sciences, University of Eastern Finland, Joensuu, Finland; 5 Departamento de Ciencias Forestales, Universidad de La Frontera, Temuco, Chile; 6 United Nations Development Programme, Costa del Este, Panama City, Panama; 7 Inter-American Development Bank, Panama City, Panama

**Keywords:** Biogeography, Forest ecology

## Abstract

We present raw tree inventory data from 41 permanent plots in mangrove forests located in two Important Bird Areas along Panama’s Pacific coast: the Bay of Parita and the Bay of Panama. The dataset provides measurements for estimating aboveground biomass and carbon stocks in living trees, following standard inventory guidelines. In addition, we present continuous maps for aboveground biomass and carbon density for the study areas. These data help address uncertainties in Panama’s mangrove carbon stock estimates, currently ranging from 25 to 71 MtC, by providing ground-based measurements and landscape-level estimates. The measurements also enable validation of landscape-scale remote sensing approaches, such as NASA’s Global Ecosystem Dynamics Investigation (GEDI), essential for regional carbon mapping. As Panama’s primary carbon sink is the Land Use, Land-Use Change, and Forestry sector, and the country has included mangrove restoration in its Nationally Determined Contributions, this dataset contributes to national climate strategies. Furthermore, it can serve as a foundation for developing a regional carbon monitoring database for Central American and Caribbean countries currently conducting mangrove inventories.

## Background & Summary

Nature-based solutions (NbS) have emerged as a key component of global climate strategies, offering cost-effective approaches to address both climate and biodiversity crises^1,2^. Among NbS, mangroves are particularly valuable ecosystems whose conservation, restoration, and management can contribute significantly to climate change mitigation and adaptation in the tropics^3^. These ecosystems are essential for preserving terrestrial and marine biodiversity while protecting coastlines^4^.

Globally, mangroves cover 14.7 Mha^5^, storing between 5.2 and 8.6 gigatons of carbon (GtC)^6^. In addition, mangroves are among the ecosystems with the highest carbon sink capacity^3^, sequestering between 9.6 to 15.8 MtC anually^6^. Despite their relevance as carbon reservoirs-sinks and biodiversity regulators, mangroves are threatened by land use change (*e.g*., urban, agriculture, aquiculture) and climate change (*e.g*., severe droughts, sea level rise)^7^. Consequently, mangrove restoration has gained prominence as a climate change mitigation strategy^8^.

In Panama, where forests cover more than 60% of the territory^9^ and the Land Use, Land-Use Change, and Forestry (LULUCF) sector serves as the sole net carbon sink maintaining the country’s carbon-negative status^9^, NbS are vital for the climate strategy. Furthermore, through millions of years, Panama has been the door, bridge, and home for thousands of species crossing between North and South America^10^. Panama, as biodiversity bridge, is an structural component of the intercontinental flyway pathways, through which millions of birds migrate every year^11–13^. Recognizing this importance, Panama has incorporated mangrove restoration into its Nationally Determined Contributions^14^.

Panamanian mangroves cover 0.187 Mha of the country; the majority is located in the Pacific, while only 3% occurs in the Caribbean^15^. Current total carbon stock estimates for these ecosystems in Panama vary widely, ranging from 25 to 71 MtC^15–18^. This uncertainty arises from multiple factors, including highly variable mangrove cover estimates; limited availability of ground data (species composition, tree height and diameter, wood specific gravity and carbon content); the inclusion of different carbon pools (living trees, dead trees, wood debris, root biomass, and soil carbon considering distinct depths (0.3 to 1 m); and methodological variations in calculating carbon stock densities (tC ha^−1^). Specifically for living tree carbon stocks, variations in sampling methodology (site selection and stratification), experimental plot design (size, shape, expansion factors), and the application of different allometric models (general or site/species-specific) significantly affect carbon stock estimates, complicating cross-study comparisons. In addition, ground data are essential for calibrating and validating remote sensing methodologies for landscape-level biomass and carbon estimates (*e.g*., NASA’s Global Ecosystem Dynamics Investigation (GEDI)) that can be used to develop more ambitious national conservation/restoration programs or develop science-based quantitative pledges for Nationally Determined Contributions. Thus, access to raw data from mangrove forest inventories combined with landscape-level continuous maps could lead to the development of a national open-access dataset allowing the standardization of the criteria for mangrove aboveground biomass estimations and regional data comparison. However, mangrove inventories present unique challenges, requiring careful timing around tidal cycles and substantial resources (*e.g*., human and financial).

This manuscript presents raw data for estimating aboveground biomass and carbon in living trees from two major Important Bird Areas (IBAs) in Panama’s Pacific region: the Bay of Parita and the Bay of Panama. The dataset encompasses measurements from 41 permanent plots established following standard guidelines as part of a regional carbon baseline assessment^19–21^. In addition, we present continuous maps for aboveground biomass and carbon density for the study areas. Together, these datasets can serve as a foundation for broader regional datasets, supporting ongoing national mangrove inventories across Central America and the Caribbean (*e.g*., the Regional Blue Carbon Monitoring, Reporting, and Verification Mechanism (MRV) funded by the UK-Blue Carbon Fund in Jamaica, Trinidad and Tobago, Suriname, Colombia, and Panama).

## Methods

This study utilized ground data collected during the “Valuing, Protecting and Enhancing Coastal Natural Capital in Panama (PN-T1233) / Blue Natural Heritage” project, implemented by Audubon Americas and funded by the UK Blue Carbon Fund through the Department for Environment, Food and Rural Affairs (DEFRA) via the Inter-American Development Bank (IDB). Field data collection was conducted between 2021 and 2024, following standardized sampling protocols for plot location, design, and measurements^19–21^. Detailed field and laboratory protocols adhered to established mangrove measurement guidelines^20,22^.

We collected data on tree diameter, height, and wood specific gravity (WSG) to estimate plot-level aboveground biomass and carbon density in living trees. This methods section describes: i) the sampling design and laboratory analyses used to generate raw data for calculating plot-level aboveground biomass density (AGBd) and aboveground carbon density (AGCd), and ii) the methodology for estimating and validating landscape-level AGBd and AGCd using remote sensing techniques.

### *In situ* sampling design

**Fig. 1:**
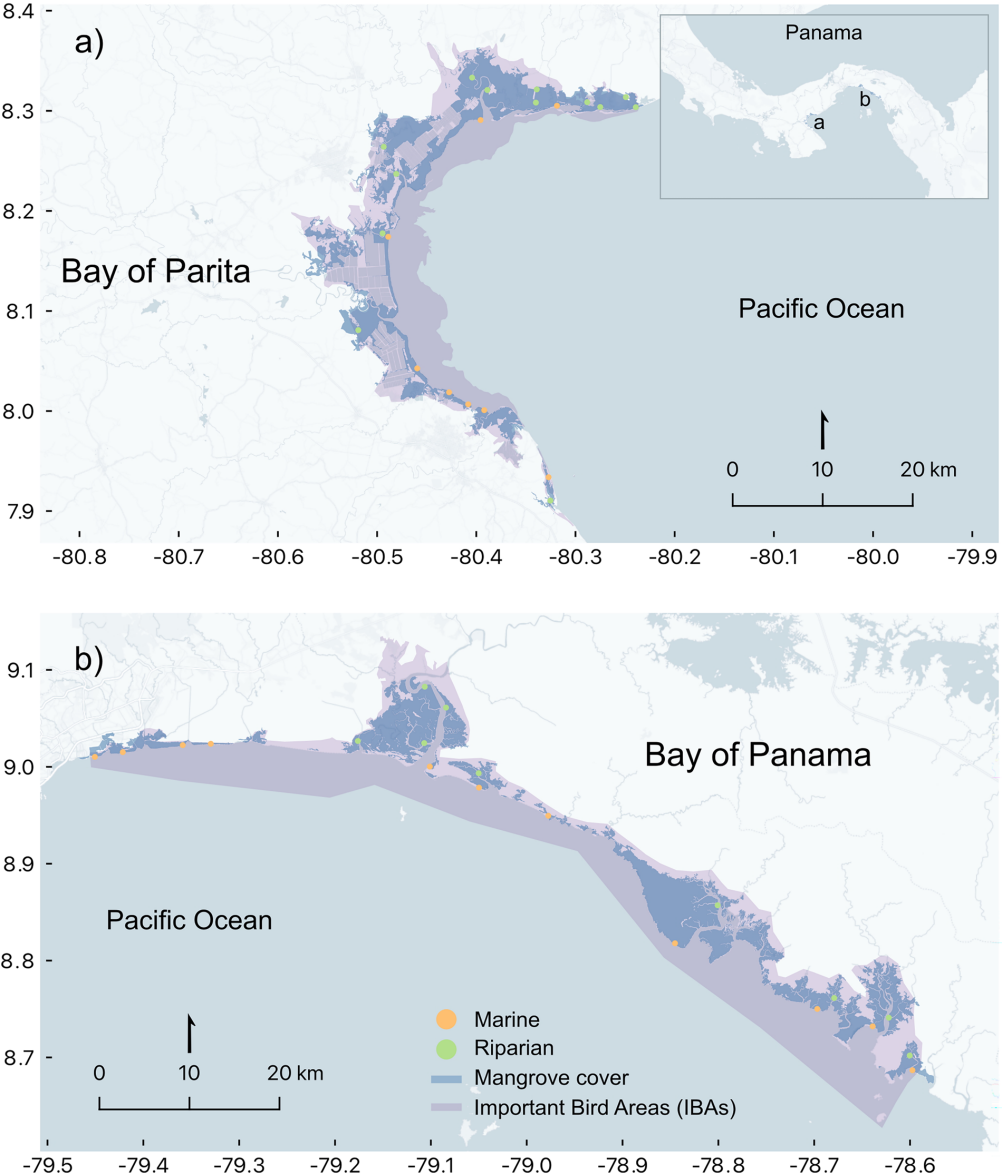
Plot locations in the study areas: (**a**) Bay of Parita and (**b**) Bay of Panama. Marine plots (orange) and riparian plots (green) are shown overlaid on mangrove cover (blue) and Important Bird Areas (IBAs, purple).

To allocate plots, we first defined the study areas based on existing shape boundaries for Important Bird and Biodiversity Areas in Panama; specifically, those located in the Bay of Parita and the Bay of Panama (Fig. 1). Next, we stratified the study areas considering two mangrove geomorphic typologies: marine (open coast) and riparian; marine mangroves were situated in open-coast, marine-edge settings, whereas riparian mangroves were located in the margins of rivers and streams^23,24^ (Fig. 1). Then, we opted for a random plot placement using a 500 by 500 m grid placed on top of the mangrove shape developed by the Ministry of the Environment, including a 180 m fringe-buffer area. Finally, we applied a criteria of a 1 km minimum distance among plots. We established 41 plots in total (Fig. 1, Table S1).

**Fig. 2:**
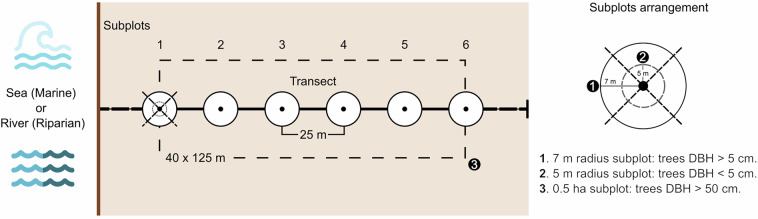
Schematic plot layout for aboveground biomass estimation in Panamanian mangroves, modified from Kauffman and Donato (2012).

Each plot consisted of a linear transect consisting of six circular subplots distributed perpendicular to the sea or river shore. Each subplot had a 7 m radius and were separated by a 25 m distance between the center of each subplot (Fig. 2)^19,21^. Whitin each subplot, a nested 5 m radius plot was also established.

## Mangroves aboveground biomass density (AGBd)

### Vegetation survey

Within each 7 m radius subplot, we identified, measured, and tagged all trees with stems >5 cm diameter at breast height (DBH at 1.3 m height). Specifically, we recorded mangrove species, measured DBH in cm using diameter tapes (Forestry Suppliers, metric fabric-283D/10 M), and measured total height (H) in m using analogue clinometers (Suunto, PM5/66PC). Within the 5 m radius subplots, we measured the same parameters for trees <5 cm DBH. Trees >50 cm DBH were measured within 40 × 125 m (0.5 ha) rectangular subplots positioned along the main transect and encompassing the 7 m radius circular subplots.

### Wood specific gravity

We collected wood samples from living trees using an increment borer (3-thread, 5.15 mm, 0.4 m; Jim-Gem, Jackson, MS, US) to estimate wood density (*i.e*., wood specific gravity, WSG). Fresh volume was measured by water displacement using 5 cm wood segments and wood dry weight was determined after drying at 100 °C (48 h) to calculate WSG_100_^25,26^. WSG was calculated as the ratio between wood dry weight and fresh volume (g cm^−3^). Using all the segments from each core, WSG was weighted by cross sectional areas to take into account the radial increase of wood volume from the tree center^26^.

### Plot-level forest biomass and carbon density

#### Living biomass

We estimated total aboveground biomass of living trees (AGB_LT_) using the general equation developed for moist mangrove forest stands^27^. The allometric equation is: AGB = 0.0509·(*ρ* ·DBH^2^ ·H), where AGB_LT_ = Aboveground biomass of living trees (kg), *ρ* = WSG_100_ (g cm^−3^), DBH = diameter at breast height (cm), and H = total height (m). For *ρ*, we used the average measured WSG_100_ value for each of the five species for which we collected wood cores (*i.e*., *Rhizophora mangle*, *Rhizophora racemosa*, *Laguncularia racemosa*, *Avicennia germinans*, *and Avicennia bicolor*). For unmeasured species, we used data available in the literature: for *Mora oleifera*, we used WSG data from the global wood density database^28,29^ and for *Pelliciera rhizophorae*, we used data from Panama’s Pacific region^30^ (Table S2). Using species specific WSG values influences the AGBd and the overall carbon stock estimates, as WSG varies significantly among mangrove species (Table S2). This impact is particularly relevant in areas inhabited by heterogeneous mangrove communities composed of species with contrasting WSG (*e.g*., *R. racemosa* with WSG ≈ 0.91 g cm^−3^ and *P. rhizophorae* with WSG ≈ 0.45 g cm^−3^).

AGB_LT_ (t, tonnes) was calculated for each tree individually. Then, AGB_LT_ of all trees within all circular (*i.e*., 6 subplots with 7 m radius and 6 nested subplots with 5 m radius plots) and rectangular subplots were summed, independently, for the total area of each subplot type to obtain the total AGB_LT_ per subplot type. The AGB_LT_ values per subplot type were then multiplied by the corresponding expansion factor to convert AGB_LT_ to Aboveground Biomass Density (AGBd, t ha^−1^). The expansion factor for each subplot type was calculated as reciprocal of the total area (ha) of each subplot type (*i.e*., 6 circular plots of 7 m radius = 923 m^2^ ≈ 0.092 ha; expansion factor = 10.82; 6 circular plots of 5 m radius = 471 m^2^ ≈ 0.047 ha; expansion factor = 21.22; and 1 rectangular plot of 40 × 125 m = 5000 m^2^ ≈ 0.5 ha, expansion factor = 2). Finally, the independent AGBd values per subplot type were added to obtain the overall plot-level AGBd.

#### Forest biomass carbon density

We determined biomass carbon content by using a biomass-carbon conversion factor of 0.5 gC g_biomass_^−1^ following standard protocols^19,20^. This factor was used to convert aboveground biomass density (t_biomass_ ha^−1^) (AGBd) into aboveground carbon density (tC ha^−1^) (AGCd).

### Remote sensing

#### Landscape aboveground biomass density data

We used Global Ecosystem Dynamics Investigation (GEDI) data for landscape-scale forest biomass density analysis (https://gedi.umd.edu/). GEDI’s satellite Light Detection and Ranging (LiDAR system), operational since late 2018, provides Level-4A products for footprint-level aboveground biomass estimation at 25 m resolution^31^. GEDI footprints are not spatially continuous and are spaced 60 m along-track and 600 m across-track producing a well-defined satellite-based gridded and geolocated spatial sampling pattern for each visit date^32^. The GEDI L4A product is derived from correlations between AGB ground data, Airborne Laser Scanning (ALS) data, and GEDI footprints developed in study areas throughout the world. First, to predict AGB using GEDI satellite data, ALS data is transformed into the relative heights (RH) measured by GEDI. Subsequently, a model relating plot-level aboveground biomass estimates and GEDI RH metrics is constructed using machine learning algorithms. This model is then applied to predict biomass for each GEDI footprint. The GEDI biomass database adopts the framework established by the Australian Terrestrial Ecosystem Research Network (TERN) Biomass Plot Library, in which above-ground biomass includes only living trees^33^.

#### Landscape-level aboveground biomass density modeling

Since GEDI footprints are not spatially continuous, it was necessary to develop a continuous landscape-level model for AGBd in the study areas. However, GEDI’s data require pre-processing before they can be used to develop a spatially continuous model. GEDI’s dataset includes quality bands that assess waveform reliability for capturing three-dimensional attributes of general GEDI products. We applied the following quality filters: degrade_flag, quality_flag, run_flag, sun elevation < 0, and GEDI’s L2A and L2B power beams filters 5, 6, 8, and 11, which are recommended for dense vegetation^31^. We selected data with degrade_flag = 0, indicating undegraded pointing and positioning information, alongside quality_flag = 1, denoting valid waveforms^34^. Additionally, we utilized run_flag = 1, which indicates good waveform fidelity for AGBd estimation^31^. We excluded GEDI data where the absolute difference between the lowest ground return from the GEDI footprint and NASA’s Digital Elevation Model (DEM) at 90 m spatial resolution from the Shuttle Radar Topography Mission (SRTM) exceeded 75 m^35^.

The landscape-level biomass density model utilized filtered GEDI Level-4A footprints as response variables and multi-source remote sensing data as predictors^36^. For predictors, we incorporated 22 variables derived from: Sentinel-1 C bands and Synthetic Aperture Radar (SAR) vegetation indices (HH band, HV band, RFDI, and RFDI SD in 100 m radius around each pixel); Sentinel-2 bands and spectral indices (B2, B3, B4, B5, B6, B7, B8, B8A, B11, B12, NDVI, NDVI SD in 100 m radius around each pixel, SAVI, NBR, and EVI); topographical descriptors of the mangroves (elevation^37^, slope^37^, and distances to sea and rivers^38^); and global canopy height^39^ (Table S3). We constructed a Random Forest machine learning model using 70% of the data for training and 30% for testing. Model performance was evaluated using R^2^ and root mean squared error (RMSE) (Fig. 6). All analyses were conducted using Google Earth Engine^40^.

## Data Records

The datasets, GeoTIFF files, and code are available at Figshare repository SDATA-25-00610^41^.

**Fig. 3:**
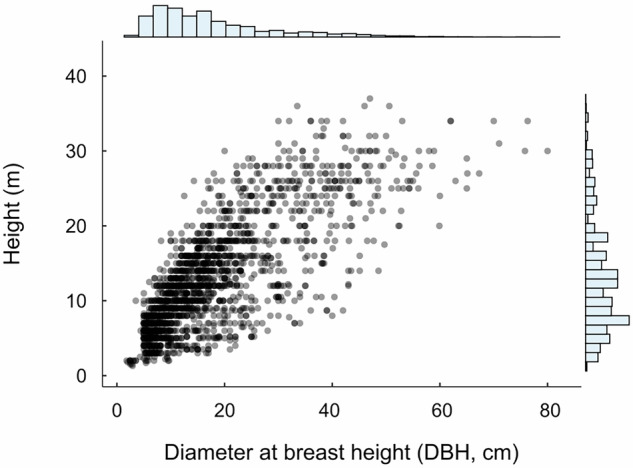
Relationship between diameter at breast height (DBH, cm) and total height (m) for all mangrove species across the Bay of Parita and Bay of Panama study areas.

**Fig. 4:**
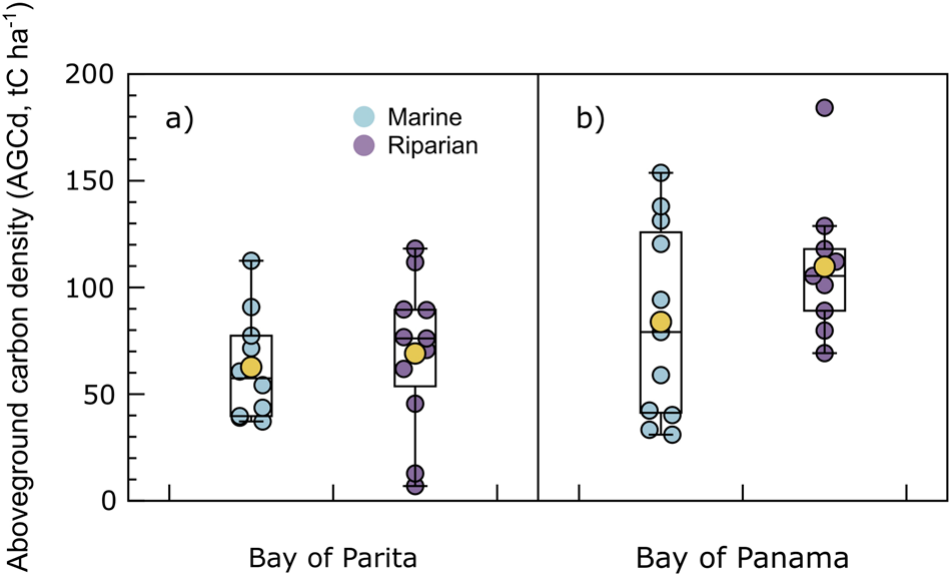
Above ground carbon density (AGCd, tC ha^−1^) for mangrove typologies (blue = marine and purple = riparian): (**a**) Bay of Parita and (**b**) Bay of Panama. Mean is presented as yellow symbols.

**Fig. 5:**
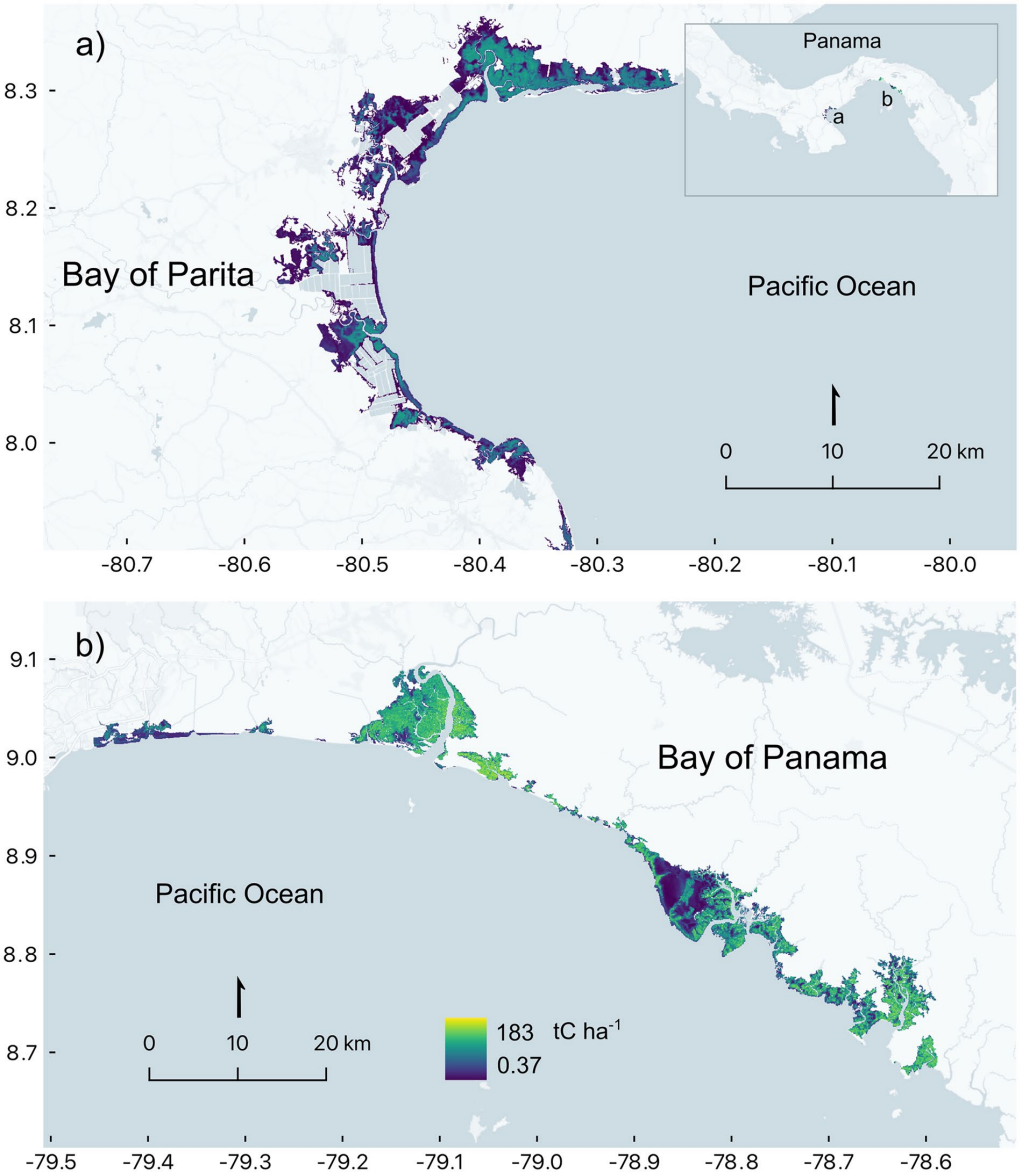
Above ground carbon density at landscape-level (AGCd, tC ha^−1^) for (**a**) the Bay of Parita and (**b**) Bay of Panama study areas.

The data records include: (**i)** the raw data to calculate the plot-level forest aboveground biomass and carbon density, (**ii)** the aboveground biomass and carbon density estimates for the 41 plots, (iii) the aboveground biomass and carbon density estimates at landscape-level, and (iv) the continuous aboveground biomass and carbon density maps. Specifically, datasets include: (i) CSV (comma separated values) file with columns representing: Registry number = continuous number for each measured tree; Study area = Bay of Parita (ParB) or Bay of Panama (PTYB); Latitude; Longitude; Typology = Marine or Riparian; C pool = carbon pool (aboveground); Sub-C pool = Living biomass; Plot = each of the 41 plots; Subplot = 1 to 6 on each plot; plotId = Plot + Subplot; Diameter class = > 5 cm DBH, <5 cm DBH, >50 cm DBH; Family = mangrove classification; Genus = mangrove classification; Species = mangrove classification; SpeciesAbb = abbreviation for each species; DBH (cm) = Tree Diameter at Brest Height measured *in situ*, expressed in cm; Height (m) = Tree total height measured *in situ* with clinometer, expressed in meters (Fig. 3). This raw data, in conjunction with the wood specific gravity (*i.e*., wood density) (Table S2), allows calculating AGBd (t_biomass_ ha^−1^) and AGCd (tC ha^−1^) at subplot or plot-level; (ii) CSV file with columns presenting:; Study area = Bay of Parita (ParB) or Bay of Panama (PTYB); Plot = each of the 41 plots; Latitude; Longitude; Typology = Marine or Riparian; AGBd (t_biomass_ ha^−1^) and AGCd (tC ha^−1^) at plot-level (Fig. 4); (**iii)** CSV file with columns presenting: Study area = Bay of Parita (ParB) or Bay of Panama (PTYB); Latitude; Longitude; AGBd (t_biomass_ ha^−1^) and AGCd (tC ha^−1^) at landscape-level from GEDI data; (**iv)** GeoTIFF files (TIFF) with AGBd (t_biomass_ ha^−1^) and AGCd (tC ha^−1^) at landscape-level for the Bay of Parita and the Bay of Panama (Fig. 5), and (**v)** ZIP files containing the code to develop the landscape-level AGBd and AGCd estimations for the Bay of Parita and the Bay of Panama (Fig. 5).

## Technical Validation

DBH and H measurements are subjected to observational and systematic errors common in forest inventories^42,43^. For mangrove H records, previous studies have estimated a 7.7% error when using trigonometric equipment such as the clinometers used for this study^44^. The technical validation of our data descriptor focused on examining diameter-height relationships to identify potential measurement biases or errors (Fig. 3). We analyzed the slenderness ratio (total height/DBH, expressed in meters) of all measurements. Of the total measurements, 6% showed extreme values: 39 measurements exhibited excessive slenderness (>150) and 75 showed very low slenderness (<30).

**Fig. 6:**
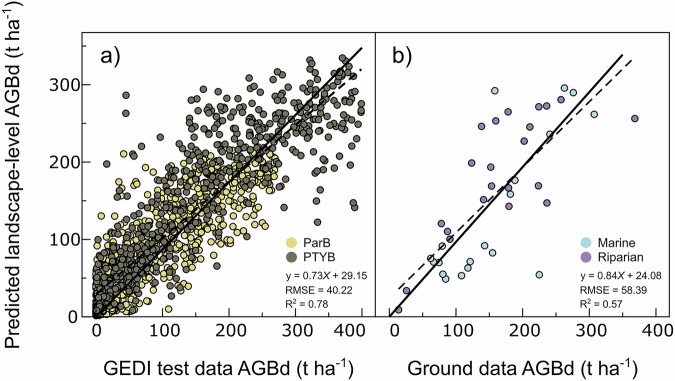
(**a**) Random Forest internal evaluation (Symbols represent study areas: yellow = Bay of Parita, dark grey = Bay of Panama; linear regression (dashed line): R^2^ = 0.78; RMSE = 40.22) and (**b**) ground/field Aboveground Biomass density (AGBd) data vs predicted landscape-level AGBd (t_biomass_ ha^−1^) (Symbols represent mangrove typology: blue = marine, purple = riparian; linear regression (dashed line): R^2^ = 0.57; Root Mean Square Error (RMSE) = 58.39 t_biomass_ ha^−1^). The solid lines represent the 1:1 relationship between the variables.

To assess landscape-level biomass estimations performance, we trained our Random Forest model using 70% of our data and evaluated it with the remaining 30%, obtaining an R^2^ = 0.78 and a Root Mean Square Error (RMSE) of 49 t_biomass_ ha^−1^ (Fig. 6a). Next, we compared our GEDI-derived AGBd model to the plot-level forest AGBd ground measurements. The relationship between modeled and ground measurements across both study areas (Bay of Parita and Bay of Panama) yielded an R² of 0.57 and a (RMSE) of 58 t_biomass_ ha^−1^ (Fig. 6b). We then computed the global Moran’s I on the residuals to test for spatial autocorrelation for the GEDI-derived AGBd residuals (N = 1 476), I = 0.05 (*P* < 0.001, permutation test) and for the plot-level AGBd residuals (N = 41), I = 0.11 (*P = *0.08). A spatial correlogram of lag-class Moran’s I showed a single peak at the maximum lag (I = 0.33, *P* = 0.156). Overall, the analysis indicated no robust long-range autocorrelation. Finally, to mitigate potential geo-location mismatch between GEDI footprints and GPS-recorded plot locations, before comparison, we smoothed both data sets with a 3 × 3 mean moving-window (*i.e*., the focal pixel plus its eight neighbors).

## Usage Notes

The aboveground biomass density (AGBd, t_biomass_ ha^−1^) and aboveground carbon density (AGCd, tC ha^−1^) estimates for living trees at plot-level were calculated using the allometric equation: AGB = 0.0509 (*ρ* DBH^2^ H). This allometric model is adequate for both moist mangrove forest stands and moist forest stands when height (H) is available^27^. The model was developed including mangrove data for trees with a maximum DBH of 42 cm, whereas the maximum DBH for moist forest trees was 156 cm^27^. Thus, we applied this model to our entire dataset, as scaling rules for mangroves have been found to be consistent with those for upland moist forest^45,46^. A carbon conversion factor of 0.5 gC g_biomass_^−1^ was applied (see Methods section). While species-specific allometric models and carbon content values would yield different AGBd and AGCd estimates, the provided raw data enables recalculation using alternative approaches. For the landscape-level AGBd and AGCd estimates, including living trees only, we applied the same carbon conversion factor (0.5 gC g_biomass_^−1^).

## Supplementary information

Supplementary information for Plot and landscape-level estimates of tree biomass and carbon stocks in Panama's mangrove Important Bird Areas

## Data Availability

R Code and files necessary to develop the landscape-level AGBd and AGCd estimations are available as part of the Figshare files^41^ (*i.e*., Bay of Parita AGBd AGCd landscape model.zip; Bay of Panama AGBd AGCd landscape model.zip).
