# Supplementary material for: Plot and landscape-level estimates of tree biomass and carbon stocks in Panama’s mangrove Important Bird Areas
Source: Sci Data. 2025 Jun 17;12:1016. doi: 10.1038/s41597-025-05354-5 (PMC12174316; doi:10.1038/s41597-025-05354-5)
Supplement: Supplementary file 1 — Supplementary information for Plot and landscape-level estimates of tree biomass and carbon stocks in Panama's mangrove Important Bird Areas [file 41597_2025_5354_MOESM1_ESM.pdf]

Supplementary information

Plot and landscape-level estimates of tree biomass and carbon stocks in Panama's mangrove Important Bird Areas

Jorge Hoyos-Santillan[1,2,3,4]*, Alejandro Miranda[5], Juliana Chavarría[2,6], Carlos Hormazabal[5], Blas Mola-Yudego[4], Esperanza González-Mahecha[7]

[1]Smithsonian Tropical Research Institute, Panama City, Panama.
[2]Audubon Americas, New York, USA.
[3]School of Biosciences, University of Nottingham, Sutton Bonington, UK.
[4]School of Forest Sciences, University of Eastern Finland, Joensuu, Finland.
[5]Departamento de Ciencias Forestales, Universidad de La Frontera, Temuco, Chile.
[6]United Nations Development Programme, Costa del Este, Panama City, Panama.
[7]Inter-American Development Bank, Panama City, Panama.

*jorge.hoyos@umag.cl

Table of contents

1. **Table S1**. Plot locations and typology in the Bay of Parita and the Bay of Panama
2. **Table S2**. Wood specific gravity (WSG) (g cm^{-3}) for dominant mangrove species in the Bay of Parita and the Bay of Panama.
3. **Table S3.** List of predictor variables used for landscape-level AGBd estimation from GEDI L4 product.

Table S1. Plot locations and typology in the Bay of Parita and the Bay of Panama

Study site[a]	Plot number	Plot Id	Latitude	Longitude	Typology
Bay of Parita	1	1 ParB	8.04263	-80.45956	Marine
Bay of Parita	2	2 ParB	8.00086	-80.39198	Marine
Bay of Parita	3	3 ParB	8.01873	-80.42740	Marine
Bay of Parita	4	4 ParB	8.30505	-80.31869	Marine
Bay of Parita	5	5 ParB	8.31367	-80.24923	Riparian
Bay of Parita	6	6 ParB	8.30376	-80.27504	Riparian
Bay of Parita	7	7 ParB	7.93383	-80.32713	Marine
Bay of Parita	8	8 ParB	7.91046	-80.32532	Marine
Bay of Parita	9	9 ParB	8.30819	-80.33992	Riparian
Bay of Parita	10	10 ParB	8.32139	-80.33904	Riparian
Bay of Parita	11	11 ParB	8.29077	-80.39571	Marine
Bay of Parita	12	12 ParB	8.32076	-80.38887	Riparian
Bay of Parita	13	13 ParB	8.33316	-80.40423	Riparian
Bay of Parita	14	14 ParB	8.30861	-80.28820	Riparian
Bay of Parita	15	15 ParB	8.08088	-80.51900	Riparian
Bay of Parita	17	17 ParB	8.17755	-80.49449	Riparian
Bay of Parita	18	18 ParB	8.23698	-80.48068	Riparian
Bay of Parita	19	19 ParB	8.17419	-80.48878	Marine
Bay of Parita	20	20 ParB	8.26409	-80.49331	Riparian
Bay of Parita	21	21 ParB	8.30362	-80.23920	Marine
Bay of Parita	22	22 ParB	8.00673	-80.40817	Marine
Bay of Panama	1	1 PTYB	8.68675	-78.59749	Marine
Bay of Panama	2	2 PTYB	8.73212	-78.63900	Marine
Bay of Panama	3	3 PTYB	8.75007	-78.69678	Marine
Bay of Panama	4	4 PTYB	8.74102	-78.62215	Riparian
Bay of Panama	5	5 PTYB	8.70199	-78.60032	Riparian
Bay of Panama	6	6 PTYB	8.76133	-78.67916	Riparian
Bay of Panama	7	7 PTYB	8.81786	-78.84524	Marine
Bay of Panama	8	8 PTYB	8.85713	-78.80078	Riparian
Bay of Panama	9	9 PTYB	8.94951	-78.97744	Marine
Bay of Panama	10	10 PTYB	8.97854	-79.04974	Marine
Bay of Panama	11	11 PTYB	8.99345	-79.05008	Riparian
Bay of Panama	12	12 PTYB	9.00025	-79.10106	Marine
Bay of Panama	13	13 PTYB	9.06110	-79.08417	Riparian
Bay of Panama	14	14 PTYB	9.08268	-79.10636	Riparian
Bay of Panama	15	15 PTYB	9.02664	-79.17615	Riparian
Bay of Panama	16	16 PTYB	9.02449	-79.10690	Riparian

Bay of Panama	17	17 PTYB	9.02378	-79.32959	Marine
Bay of Panama	18	18 PTYB	9.01026	-79.45060	Marine
Bay of Panama	19	19 PTYB	9.01514	-79.42142	Marine
Bay of Panama	20	20 PTYB	9.02237	-79.35887	Marine

[a]Bay of Parita = ParB; Bay of Panama = PTYB

Table S2. Wood specific gravity (WSG) (g cm^{-3}) for dominant mangrove species in the Bay of Parita and the Bay of Panama. Data corresponds to mean ± SE.

Species	n	WSG_{60}[a]	WSG_{100}[b]	WSG Bibliography
Laguncularia racemosa (Lr)	6	0.65 ± 0.04	0.65 ± 0.03	
Avicennia germinans (Ag)	17	0.76 ± 0.02	0.74 ± 0.02	
Avicennia bicolor (Ab)	7	0.79 ± 0.01	0.78 ± 0.01	
Rhizophora mangle (Rm)	7	0.91 ± 0.02	0.89 ± 0.02	
Rhizophora racemosa (Rr)	2	0.92 ± 0.01	0.91 ± 0.01	
Pelliciera rhizophorae (Pr)[c]	–			0.45 ± 0.02
Mora oleifera (Mo)[d]	4			0.81 ± 0.01

[a,b]Wood specific gravity was measured at 60 and 100 °C following ForestGeo protocols.

[c]Dangremond, E. M. & Feller, I. C. Functional traits and nutrient limitation in the rare mangrove Pelliciera rhizophorae. *Aquabot* **116**, 1–7 (2014).

[d]Chave, J. *et al.* Regional and phylogenetic variation of wood density across 2456 neotropical tree species. *Ecol. Appl.* **16**, 2356–2367 (2006).

Table S3. List of predictor variables used for landscape-level AGBd estimation from GEDI L4 product.

**RADAR**	**Source**	**Process**	**Resolution**
HV and HV bands (DN)	Global Palsar-2 (GEE)	Median annual value in the period 2019-2024.	25 m
Radar Forest Degradation Index (RFDI)	Global Palsar-2 (GEE)[1]	RFDI = (HH - HV) / (HH + HV)	25 m
VV and VH bands (dB)	Sentinel-1 SAR GRD: C-band (GEE)	Mean value from the month of GEDI acquisition month.	10 m
**Earth surface reflectance information**			
B2, B3, B4, and B8 bands	Harmonized Sentinel-2 MSI: MultiSpectral Instrument, Level-2A (GEE)	Mean value from the GEDI acquisition month.	10 m
B5, B6, B7, B8A, B11, B12	Harmonized Sentinel-2 MSI: MultiSpectral Instrument, Level-2A (GEE)	Mean value from the GEDI acquisition month.	20 m
(Normalized difference vegetation index) NDVI	Harmonized Sentinel-2 MSI: MultiSpectral Instrument, Level-2A (GEE)	NDVI = (B8 – B4) / (B8 + B4)	10 m
Soil Adjusted Vegetation Index (SAVI)	Harmonized Sentinel-2 MSI: MultiSpectral Instrument, Level-2A (GEE)	SAVI = (B8 – B4) / (B8 + B4 + 0.428) * (1.428)	10 m
Normalized Burned Ratio Index (NBR):	Harmonized Sentinel-2 MSI: MultiSpectral Instrument, Level-2A (GEE)	NBR = (B8 – B12) / (B8 + B12)	20 m
Enhanced Vegetation Index (EVI)	Harmonized Sentinel-2 MSI: MultiSpectral Instrument, Level-2A (GEE)	EVI = 2.5 * (B8 – B4) / ((B8 + 6 * B4 – 7.5 * B2) + 1))	10 m
Canopy Height	Global canopy top height for the year 2020 at 10 m spatial resolution[2].	Data is available at: https://nlang.users.earthengine.app/view/global-canopy-height-2020	10 m
**Topography and geography**			
Distance to the coast (km)	Developed in this research	Calculated in GEE with the Forest Cover and Land Use 2021 vector data for Panama[3] through the Euclidean distance to the coastal line. Vector data available at: https://www.arcgis.com/home/item.html?id=79d799667f994308869d974cf9bee7ef	30 m
Elevation (m.a.s.l.)	Copernicus DEM GLO-30 (GEE)	Extraction of direct data available at: https://developers.google.com/earth-engine/datasets/catalog/COPERNICUS_DEM_GLO30	30 m
